# Concomitant Occurrence of Peripheral Neuropathy and Vision Loss Due to Multivitamin Deficiency After Bariatric Surgery

**DOI:** 10.7759/cureus.59959

**Published:** 2024-05-09

**Authors:** Muhammad J Khalid, Muhammad A Ayub, Saurabh Kataria, Michael Hebert, Arvin Parvathaneni

**Affiliations:** 1 Neurology, Louisiana State University Health Sciences Center, Shreveport, USA

**Keywords:** vision loss, suplementation, gastric bypass surgery, nutritional deficiency, neuropathy

## Abstract

Vitamin deficiencies, especially after Bariatric surgery, are common and, when not properly addressed, can lead to debilitating complications. Bariatric procedures, to variable degrees, alter the anatomy and physiology of the gastrointestinal; this alteration makes these patients more susceptible to developing nutritional deficiencies. Peripheral neuropathy is one of the complications that can arise from nutritional deficiencies, and it can cause severe functional impairment. Vision loss is a relatively uncommon complication after weight loss procedure. Changes in the retinal nerve fiber layer, choroidal thickness, and visual fields due to hypovitaminosis result in nutritional optic neuropathy and retinopathy. The main retinal complication is nyctalopia (night blindness), which is caused by vitamin A deficiency. We present a case of concomitant peripheral neuropathy and vision loss secondary to reduced levels of multiple vitamins following gastric bypass surgery. This case highlights the need for regular vitamin level monitoring and appropriate replenishment in patients after bariatric surgery to prevent significant morbidities.

## Introduction

Bariatric surgery (BS) has been demonstrated to achieve meaningful weight loss in many patients since its inception in 1950. BS is becoming increasingly more popular, and the American Society for Metabolic and Bariatric Surgery estimates over 260,000 bariatric surgeries will be performed in 2021 [1]. However, it can cause a significant loss of micronutrients if not addressed appropriately. Neurological complications are the main manifestations of this loss. Some studies estimate that 4.6% of patients reported to have neurological complications. The constellation of neurological complications included chronic and subacute peripheral neuropathy (52%), acute peripheral neuropathy (4%), burning feet (9%), meralgia paresthetica (9%), myotonic syndrome (4%), posterolateral myelopathy (9%), and Wernicke encephalopathy (9%) [2]. Thaisetthawatkul et al. observed that 8.6% of his study population developed neurological complications [3]. Of these 48 patients, 23 (48%) developed mononeuropathies with carpal tunnel syndrome, the most common adverse effect accounting for 74% of the total. Peripheral neuropathies were observed in 20 (42%), plexopathies in four (17%), and myopathy in one (4%) [3]. Bariatric surgeries can induce ophthalmic complications that can affect almost every component of the optic system: the conjunctiva, the cornea, the retina, and the optic nerve, depending on which nutrient is deficient. Vitamin A metabolism can be impaired before BS due to the existence of nonalcoholic fatty liver disease, which can occur in 71 to 88 % of obese patients. Surgery further potentiates its deficiency. The main ophthalmic complication is night blindness (2.8%) [2]. We present a case of a patient with concomitant occurrence of peripheral neuropathy and vision loss after bariatric surgery due to vitamin deficiency.

## Case presentation

A 47-year-old African American female presented for evaluation of progressive weakness and vision loss. She stated that her symptoms started a year ago and were progressively worsening. She mentioned that she started using a wheelchair to ambulate as she had a couple of falls. The patient further elaborated that her weakness and numbness started in her feet and ascended gradually to involve her upper extremities. She denied tingling but acknowledged having paroxysmal lancinating pain in her legs. Her vision started becoming blurry over a period of a week. Upon enquiring about further history, she reported that a few months before, she started to develop these symptoms. She underwent a Roux-en-Y gastric bypass surgery for weight loss seven months before she was presented with symptoms. Her body mass index (BMI) before surgery was 44.5 kg/m^2 ^which went down to 36.7 kg/m^2^ after the intervention. After the surgery, she was having regular follow-ups and was placed on oral over-the-counter multivitamins supplementation, which she reported taking consistently. 

A neurological exam revealed an alert and oriented lady with symmetrical round and reactive pupils to light and intact accommodation, intact extraocular movements, and a decrease in visual acuity on Snellen’s chart, which was 20/800 in the right eye and 20/400 in the left eye. Fundus examination showed mild asymmetrical optic disc cupping. She had normal muscle tone without atrophy and fasciculations. Muscle strength was graded 5/5 throughout, except for plantar flexion, which was 4/5 bilaterally. There was a severe symmetrical sensory loss to light touch and pinprick, which was more prominent distally. Vibrations and proprioception were also significantly decreased at the toes. Areflexia was noted in patellar and ankle reflexes. Triceps, biceps, and brachioradialis reflexes were reduced to 1+. Plantar reflexes were down and there was a negative Hofmann sign. Coordination could not be accurately assessed due to severe vision loss and subjective weakness. Her investigations revealed vitamins B1, B6, and A deficiency (Table 1). 

**Table 1:** Vitamins B1, B6, and A deficiency after gastric bypass surgery

Tests	Results	Reference Range
Thiamine	22 ug/L	38-122 ug/L
Vitamin B6	2 ug/L	5-50 ug/L
Retinol, serum	14.0 mcg/dL	(32.5-78.0) mcg/dL
Vitamin E	9.8 mg/L	5.5-17 mg/L
Vitamin B12	1793 pg/ml	210-950 pg/ml
Folate	12.2 ng/ml	4.0-24 ng/ml

**Figure 1:**
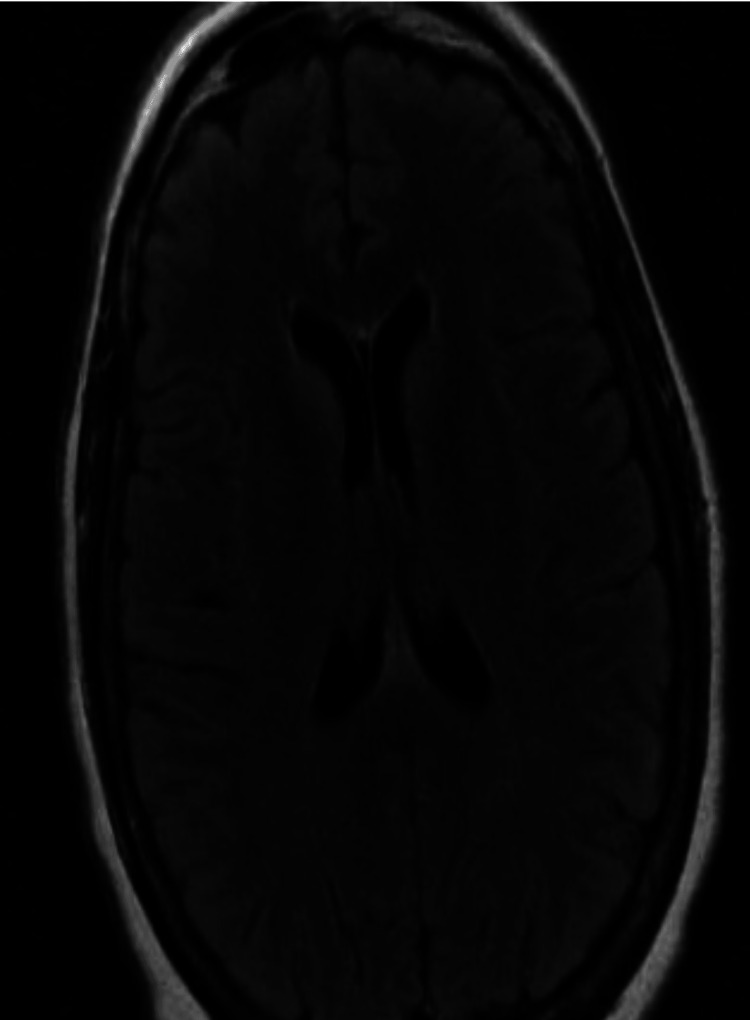
MRI of the brain (without contrast) MRI Brain did not reveal demyelinating plaques or ischemic changes.

**Figure 2:**
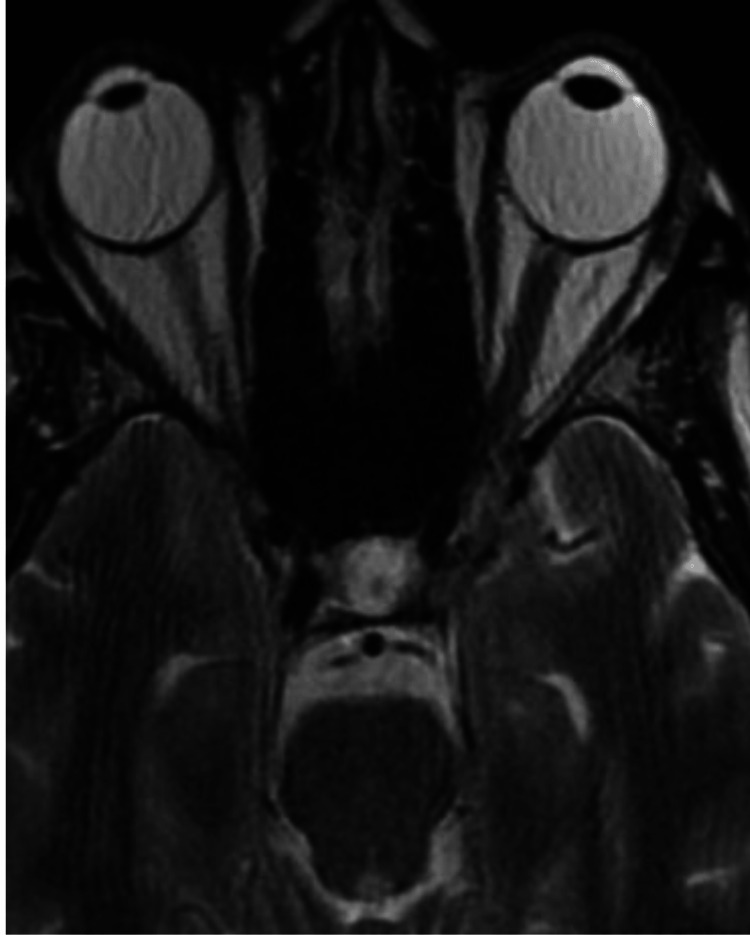
MRI of the brain (with contrast) MRI orbits were unremarkable for flattening of the posterior globe, optic nerve atrophy, or demyelination.

Other infectious and metabolic investigations for peripheral neuropathy and vision loss were unremarkable, including diabetes, HIV, syphilis, paraneoplastic, and vasculitis. Electromyography revealed fibrillations and positive waves. Nerve conduction studies demonstrated increased distal latencies, a decrease in amplitude in compound motor action potential (CMAP), and sensory nerve action potential (SNAP) in the median nerve, which was consistent with severe axonal damage. She was evaluated by ophthalmology for vision loss and was found to have no other significant exam abnormalities other than decreased visual acuity and mild optic cupping. Magnetic resonance imaging (MRI) with and without contrast of brain and orbits were unremarkable for demyelinating plaques, optic disc, or optic nerve pathology (Figures 1, 2) 

The etiology of the patient’s presentation was suspected to be nutritional deficiencies secondary to malabsorption from BS. She started taking vitamin supplementation, including oral thiamine 100 mg three times daily for three days, followed by 100 mg once daily, oral pyridoxine 100 mg once daily, and oral vitamin A 10,000 units daily for six months. She was discharged to a skilled nursing facility where she had three hours of physical therapy daily for two months. Later, she did outpatient physical therapy three times a week. She started ambulating with the help of a cane within six months. Her visual acuity improved to 2/100 in both eyes on a follow-up visit after six months.

## Discussion

BS is a very effective procedure to help decrease weight, but it can cause other potentially worse outcomes. Studies have reported that African Americans will have more chances to develop vitamin A, D, and B1 deficiency after one year, and B1 and B6 deficiency after two years. Vitamin deficiencies after laparoscopic Roux-en-Y gastric bypass are more common and involve more vitamins, even those that are water-soluble [4]. 

Most patients undergoing BS have low levels of nutrients before the procedure, and these levels are reduced further after the procedure, as reported in the literature. Almost 38% of patients with morbid obesity had low serum iron, 24% had low serum folate, and 11% had low serum vitamin B12. Another study revealed a frank deficiency of vitamin D (< 20 ng/mL) and iron (< 35 ug/dL for females and < 50 ug/dL for males) in 71.4% and 36.2% of 58 BS candidates [5,6]. Nutrient deficiency in the setting of BS is due to a decrease in the absorption area of the gastrointestinal tract. Micronutrients have specific destinations in the tract to be absorbed. For example, iron absorbs duodenum, folic acid in the jejunum, and b12 in the ileum. The removal of a portion of the tract results in a deficiency of specific nutrients [7]. 

Thiamine deficiency results in beriberi, encephalopathy, and Wernicke-Korsakoff syndrome. Corneal vascularization and blindness happen in riboflavin deficiency. Pellagra is a triad of dementia, diarrhea, and dermatitis caused by niacin deficiency. Peripheral neuropathy is caused by cobalamin, pyridoxine, copper, and tocopherol deficiency. Ataxia, cerebellar degeneration, and dementia are also expected in nutritional deficiencies. However, there is no literature regarding the concomitant presence of peripheral neuropathy and vision loss in the same patient due to multiple vitamin deficiencies. Our patient was presented with a simultaneous occurrence of peripheral neuropathy and vision loss due to multiple vitamin deficiencies. Like peripheral neuropathy, vision loss is not limited to only one micronutrient. Vision loss in patients after bariatric surgery is a recognized complication [8]. It can occur because of vitamin A deficiency, which typically manifests as nocturnal vision with xeropthalmia, along with cutaneous features such as dry skin and hair. Normally, vitamin A deficiency is seen in third-world countries and people with defects in liver storage. However, a deficit in vitamin A was seen in 13% of examined patients in the first postoperative year [9]. Copper deficiency is another cause of vision loss. The main resorption sites are the stomach and duodenum. Around 10-12% of patients undergoing BS exhibited a deficit in copper [10]. Wernicke disease is another spectrum of diseases, including blindness because of thiamine deficiency after bariatric surgery [11]. 

Besides adequate replenishment and maintenance of micronutrients, dietary counseling is often helpful to ensure compliance with proper diet and supplementation. The benefit of dietary counseling was established by a study in which patients who underwent BS were randomized to either dietary counseling or standard of care for the first four months after surgery. Patients in the dietary counseling group reported significant positive changes in several eating behaviors believed to be important for successful outcomes after surgery [12]. 

## Conclusions

It is not uncommon to develop nutritional deficiency after BS. Several factors play a role in causing abnormally lower levels of vitamins and minerals. These include decreased appetite, loss of a significant portion of the gastric absorption tract, and pre-existing nutritional problems before the procedure. Nutritional deficiency and its associated unfavorable outcomes can be prevented if proper replacement of nutrients is done both before and after the procedure. Vitamin levels should be monitored regularly and replenished accordingly before and after gastric bypass surgery to prevent neurological complications.
